# Investigating the Effect of Topical Dexamethasone at the Site of Thyroid Surgery (on the Parathyroids) on Blood Calcium Levels after Surgery, Compared to the Control Group in Patients who Underwent Total Thyroidectomy

**DOI:** 10.31661/gmj.v13i.3349

**Published:** 2024-05-04

**Authors:** Mohammad Shirkhoda, Seyed mohammad Hossein Mousavi, Mohammad Shafiee, Amirmohsen Jalaeefar, Iraj Harirchi

**Affiliations:** ^1^ Cancer Research Center, Cancer Institute of Iran, Tehran University of Medical Sciences, Tehran, Iran; ^2^ Department of Radiology, Shariati Complex Hospital, Tehran University of Medical Sciences, Tehran, Iran

**Keywords:** Thyroidectomy, Dexamethasone, Calcium

## Abstract

Background: Considering the side effects of systemic injection of dexamethasone, we decided to investigate the effect of local spraying of dexamethasone in the thyroid site after thyroidectomy on postoperative calcium levels in patients who undergo total thyroidectomy. Materials and Methods: The present investigation was a randomized clinical trial of dexamethasone on 60 patients undergoing thyroidectomy, who were assigned to two groups, namely the dexamethasone and control groups, each including 30 patients. Age, gender, type of underlying pathology was recorded in medicine. Also, the blood calcium level was recorded before surgery, 6, 24 and 48 hours after thyroidectomy. Statistical analysis was performed with SPSS version 20 using tests including t-test, paired t-test, Chi-squared, and repeated measures ANOVA. Results: On average, the 24-hour postoperative calcium level was found to be significantly different between the two groups (P0.001). Nevertheless, no such difference was found to exist between the average calcium level 6 and 48 hours after the operation (P0.05). Additionally, we noted a marked difference between the average blood calcium level, before and 48 hours after the operation, in both investigated groups (P0.05). Conclusion: Accordingly, local spraying of dexamethasone in the thyroid site, after thyroidectomy, is effective on the calcium level after the operation and reduces the rate of hypocalcemia 24 hours after the operation.

## Introduction

Thyroidectomy is the complete or partial removal of the thyroid gland. Thyroidectomy is done to treat thyroid disorders such as: thyroid cancer, goiter, hyperthyroidism. The amount of thyroid gland removal during surgery depends on the reason for the patient’s visit [1][2]. Complications of any surgical operation contains an important part of the quality of that operation and these complications are different depending on the surgeon and reporting centers [3]. Important early complications include: recurrent nerve damage and damage to parathyroid and hypocalcemia, bleeding from the wound site and hematoma formation, and tracheomalacia, and late complications include hypothyroidism and permanent hypocalcemia [4].

The incidence of post-thyroidectomy hypocalcemia is estimated to be 2.5%, which is mostly temporary and is created by thyroid traction and thyroid and parathyroid arteries and damage to the parathyroid vessels during thyroidectomy and causes transient hypocalcemia [5]. Permanent hypocalcemia occurs after removal of parathyroid during total thyroidectomy. Permanent hypocalcemia occurs when the patient’s need to receive calcium lasts for more than 6 months (6).

Several studies have been conducted on the methods of predicting hypocalcemia after thyroid surgery, but it is still impossible to exactly predict it [8][9]. For this reason, many investigative attempts have evaluated the effect of different drugs on transient hypocalcemia to mitigate the possibility of temporary hypocalcemia and duration of hospitalization, while promoting the likelihood of patient discharge within the first days following surgery [10][11]. One of the drugs that its effect on reducing the likelihood of post-operative hypocalcemia is discussing, is corticosteroids such as dexamethasone. According to a number of clinical investigations involving different major and minor surgical methods, pretreatment with glucocorticoids may regulate the mechanisms responsible for post-operative inflammatory response, resulting in dampened post-operative inflammatory response, side effects, duration of hospitalization and the risk of transient hypocalcemia [12][13].

Considering the side effects of systemic injection of glucocorticoids, we sought to investigate potential post-operative effects of dexamethasone when sprayed locally at the thyroid in patients undergoing thyroidectomy to determine how this intervention may affect the post-operative calcium levels.

## Materials and Methods

Ethical Considerations

The protocol for the current study was approved by the Research Ethics Committee of Tehran University of Medical Sciences, Tehran, Iran (Approval ID: IR.TUMS.IKHC.REC.1401.452). After being given full information regarding the procedures, the participants were asked to provide written informed consent. To increase transparency, the present trial was also registered at Iran Clinical Trial Registry (IRCT20230323057764N1), and was conducted in full adherence with the Declaration of Helsinki.

Sampling and Randomization

We predetermined our sample size based on the findings of another study with similar premise and methodology [14] and also using the following equation and setting a significance level of 5% and a test power of 80%, and considering a standard deviation of 0.87 for calcium after the intervention and in considering an error of 0.6 regarding mean calcium between the control and intervention groups. Our calculations suggested a sample size of 32 in each group

**Figure:**
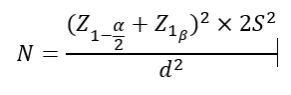


In this study, the sample size is 60 people, which is less than 100. If people are divided between 2 groups by simple random method. According to the rule of randomization of taps and lines, the number of taps and lines may not be equal in two groups. Therefore, the Block-Randomization method is used. The sample size calculated in this study is 30 people in each group. Therefore, 10 blocks of 4 people are formed and 5 blocks are randomly assigned to each group. As a result, the balance between the two groups is allocated in the form of 30 people to one group and another 30 people to the second group. A simple random method is used to assign people to each block. It means that people are placed in one of these blocks based on a table of random numbers

## Study Design

**Figure-1:**
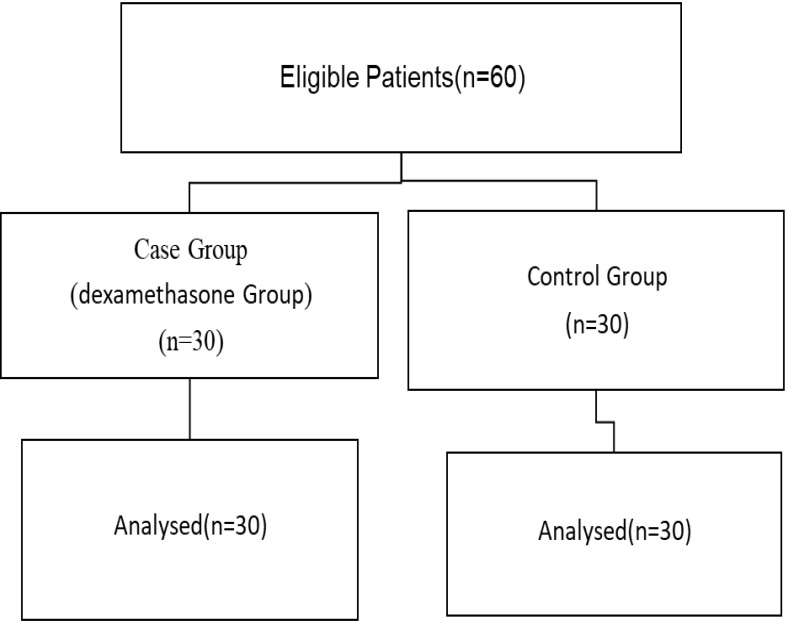
Figure 1. Consort Flow Diagram

Based on before-and-after design, the present investigation was a randomized clinical trial on a population of patients recruited by Tehran University of Medical Sciences (Cancer Institute Center).

Criteria for inclusion: All candidate patients for total thyroidectomy referred to Imam Khomeini Hospital and underwent surgery at Cancer Institute Center.

Criteria for exclusion: Patients with pathology other than cancer, patients who are candidates for subtotal or near-total thyroidectomy and unwillingness to participate (Figure-1).

After finalizing the list of included participants, we proceeded with assigning them to two groups of 30 individuals, each with their own random designation. In the intervention group, after thyroidectomy in the same operating room, an ampoule of dexamethasone (8 mg-2 ml) was sprayed in the thyroidectomy site, and in the second group (control group), no special action was taken.

Information such as age, gender, type of underlying pathology, and blood calcium level was gathered using questionnaire as well as the available medical records prior to the surgery. After surgical intervention, blood calcium levels were measured and recorded on three different timepoints, namely 6 hours, 24 hours and 48 hours after thyroidectomy [15], which was prospectively used for comparative analysis.

Statistical Analysis

All data were statistically analyzed using IBM SPSS software version 20 (SPSS, Chicago, IL). For quantitative variables with normal distribution, we determined mean ± SD. To compare data between the two groups, we used the chi-squared test and T-test. As for the means of quantitative data, we used the paired T-test for performing preoperative vs. postoperative comparisons. To assess temporal changes in the value of quantitative variables, we used the repeated measures ANOVA test. The threshold of statistical significance was presumed to correspond to a P value of less than 0.05.

## Results

**Table:** Table1. Baseline characteristics of the intervention and control groups.

Variable		Intervention (n=30)	Control (n=30)	P-value
Age (mean ±SD)		42.10±10.58	40.87±12.21	0.677*
Sex	Male	8 (26.7 %)	9 (30 %)	0.774**
	Female	22 (73.3 %)	21 (70 %)	
Underlying pathology	PTC	25 (83.3 %)	24 (80 %)	0.642**
	FTC	2 (6.7 %)	4 (13.3 %)	
	MTC	3 (10 %)	2 (6.7 %)	

^*^ T-test ^**^> Chi-Squared Test **MTC:** Medullary Thyroid Carcinoma **PTC:** Papillary Thyroid Carcinoma **FTC:** Follicular Thyroid Carcinoma

**Table:** Table2. Mean blood calcium level at specified times in intervention and control groups.

Time	Intervention	Control	P-value
Before thyroidectomy	9.91±0.67	9.83±0.56	0.619*
6 hours after thyroidectomy	9.75±0.54	9.54±0.54	0.136*
24 hours after thyroidectomy	9.51±0.58	8.86±0.52	<0.001*
48 hours after thyroidectomy	8.95±0.51	8.79±0.54	0.243*
Difference mean before thyroidectomy & 48 hours after thyroidectomy	0.96±0.16	1.04±0.02	--
P-value	<0.001**	<0.001**	

^*^ T-test ^**^ Chi-Squared Test

The present investigation included 60 participants, 17 (28.3%) male and 43 (71.7%) female, whose mean age was 41.48 ± 11.39 years. We did not notice any difference of potential significance in demographic information including age, sex and type of underlying pathology between the two groups (Table-1).

All participants were evaluated prior to thyroidectomy and followed-up at 6 hours, 24 hours and 48 hours after thyroidectomy.

The preoperative and postoperative (6 hours, 24 hours and 48 hours) mean blood calcium levels are listed in Table-2.

Analysis using the T-test indicated a significant increase in the average blood calcium level at 24 hours following thyroidectomy in the intervention group, as compared to the control group. The results of paired T-test suggested that the mean blood calcium level had markedly decreased in both groups 48 hours after thyroidectomy, in comparison to their corresponding values prior to thyroidectomy.

To assess the trend of changes in the mean blood calcium level during the before thyroidectomy, 6 hours, 24 hours and 48 hours after thyroidectomy, we used the repeated measures ANOVA test (Figure-1), which returned significant findings in the case of the mean blood calcium level between the two groups (P≤0.001).

## Discussion

Based on the findings of the present study, the mean age of our participants was 41.48, which was highly comparable to the mean age of 38.9 reported by Adeel et al. (14). Totally, it seems that the highest age for performing thyroidectomy is related to the 4th and 5th decades of life; although the type of underlying pathology is also effective in its occurrence; so that in the current study, 81.6% of the studied patients had PTC, 10% had FTC and 8.4% had MTC. The results of the study on gender frequency distribution showed that 28.3% of the studied patients were male and 71.7% were female. In a recent investigation that was published in 2021, 81.2% of the studied patients were women and 18.8% were men [15]; which was in agreement with our findings. Generally, as previous studies have shown, the frequency of performing total thyroidectomy is more in women than in men; the reason for this issue can be referred to the higher incidence of PTC (as the most common cause of thyroidectomy) in women than in men.

But, regarding the effect of dexamethasone on blood calcium level after performing thyroidectomy, we noticed a statistically marked difference between the average calcium level 24 hours after the surgery between the two studied groups; and calcium level in the dexamethasone group was considerably higher when compared with the control group. However, no such difference was found between the average calcium level, 6 and 48 hours after the surgery; there was also a statistically significant difference between the average blood calcium level before and 48 hours after the surgery in both studied groups, that considering the total thyroidectomy, the significant decrease in calcium level after surgery compared to before surgery can be justified. According to Adeel et al., the incidence of hypocalcemia within the first 24 hours after thyroidectomy is 24.4%, with 9.4% of patients exhibiting symptoms. As for the placebo group, the incidence of hypocalcemia within 3 days from thyroidectomy was only 4.2%; while none of the patients in the dexamethasone group had hypocalcemia. The frequency of symptomatic hypocalcemia in dexamethasone group patients was significantly lower than placebo group patients [15]. In another study conducted by Mario, it was found that the frequency of transient hypocalcemia in the placebo and dexamethasone group, while being substantial, was 37% and 12.8%, respectively [16]. That the results of these two studies were in agreement with the findings of the current study about the effect of dexamethasone on hypocalcemia after thyroidectomy.

Nevertheless, another investigation in 2019 suggested that a total of 39.1% of patients had post-operative transient hypocalcemia. Treatment with dexamethasone was not found to be associated with significantly lesser occurrence of transient post-operative hyperparathyroidism and hypocalcemia. In addition, the likelihood of post-operative symptomatic hypocalcemia in the control group (68%) was over two times higher than that of the dexamethasone group (32%). Though, statistically, this was not deemed as significant [14].

## Conclusion

Accordingly, local spraying of dexamethasone in the thyroid site, after thyroidectomy, is effective on the calcium level after the operation and reduces the rate of hypocalcemia 24 hours after the operation; albeit no significant effect on the reduction of hypocalcemia rate, at 6 hours and 48 hours after thyroidectomy. The most important limitation of the present study was the lack of PTH measurement and calcium measurement for three times.

## Acknowledgement

This research is based on research project approved by Deputy of Research of Tehran University of Medical Sciences (TUMS). The authors would like to thank all of the patients participating in the study for their cooperation and contribution.

## Conflict of Interest

The authors declare that there is no conflict of interest in the publication of this paper.
